# Benefits of calorie restriction in mice are mediated via energy imbalance, not absolute energy or protein intake

**DOI:** 10.1007/s11357-024-01166-4

**Published:** 2024-06-08

**Authors:** Daniel L. Smith, Sharon E. Mitchell, Maria S. Johnson, Victoria K. Gibbs, Stephanie Dickinson, Beate Henschel, Rui Li, Kathryn A. Kaiser, Daniella E. Chusyd, Andrew W. Brown, David B. Allison, John R. Speakman, Tim R. Nagy

**Affiliations:** 1 Department of Nutrition Sciences, University of Alabama at Birmingham, Birmingham, AL USA; 2 Nutrition Obesity Research Center, University of Alabama at Birmingham, Birmingham, AL USA; 3 Integrative Center for Aging Research, University of Alabama at Birmingham, Birmingham, AL USA; 4 Nathan Shock Center of Excellence in the Basic Biology of Aging, University of Alabama at Birmingham, Birmingham, AL USA; 5 Diabetes Research Center, University of Alabama at Birmingham, Birmingham, AL USA; 6 School of Biological Sciences, University of Aberdeen, Aberdeen Scotland, Scotland, UK; 7 Department of Clinical and Diagnostic Sciences, University of Alabama at Birmingham, Birmingham, AL USA; 8 Department of Epidemiology and Biostatistics, School of Public Health-Bloomington, Indiana University, Bloomington, IN USA; 9 Department of Health Behavior, University of Alabama at Birmingham, Birmingham, AL USA; 10 Department of Environmental and Occupational Health, School of Public Health-Bloomington, Indiana University, Bloomington, IN USA; 11 Department of Applied Health Science, Indiana University School of Public Health-Bloomington, Bloomington, IN USA; 12 Department of Biostatistics, University of Arkansas for Medical Sciences, Little Rock, AR, USA; Arkansas Children’s Research Institute, Little Rock, AR USA; 13 Shenzhen Key Laboratory for Metabolic Health, Center for Energy Metabolism and Reproduction, Shenzhen Institutes of Advanced Technology, Chinese Academy of Sciences, Shenzhen, China; 14 Institute of Health Sciences, China Medical University, Shenyang, Liaoning, China; 15 Institute of Genetics and Developmental Biology, Chinese Academy of Sciences, Beijing, China

**Keywords:** Longevity, Aging, Ambient temperature, Thermoneutral, Calorie restriction, Body composition

## Abstract

Caloric restriction (CR) results in reduced energy and protein intake, raising questions about protein restriction’s contribution to CR longevity benefits. We kept *ad libitum* (*AL*)–fed male C57BL/6J mice at 27°C (AL27) and pair-fed (PF) mice at 22°C (22(PF27)). The 22(PF27) group was fed to match AL27 while restricted for calories due to cold-induced metabolism. The 22(PF27) mice had significantly lower body weight, lean mass, fat mass, leptin, IGF-1, and TNF-α levels than AL27 mice (*p*<0.001 for all). Manipulations over ~11 weeks resulted in significant differences in body temperature, physical activity, and expression of key genes linked to hunger in the hypothalamus. Survival was significantly greater in 22(PF27) compared to AL27 overall (*p*<0.001). CR in the context of equivalent energy and protein intake resulted in hormonal, metabolic, and physiological benefits and extended longevity. Hence, energy imbalance, rather than low energy or protein intake per se, mediates the benefits of CR.

**Supplementary Information:**

The online version contains supplementary material available at 10.1007/s11357-024-01166-4.

## Introduction

Calorie restriction (CR) remains the most robust means to delay aging and increase lifespan in laboratory organisms. CR is most often implemented by reducing daily food provision. This generates a mismatch between energy requirements and energy intake [1] but also results in a proportional reduction of all macronutrients (i.e., carbohydrates, fats, and proteins). Animals respond to this mismatch by withdrawing stored energy reserves, reducing energy expenditure, altering metabolites, and activating stress response pathways [2, 3]. Multiple hypotheses have been proposed to explain the benefits of CR [4, 5], although agreement on one mechanism remains elusive. Transient energy imbalance during periods of CR may be a primary driver of long-term disease protection, health, and longevity benefits [1, 4–6]. Because the energy imbalance is only transitory for most of their lives, individuals under CR are in balance but feeding at a lower level of absolute intake. An alternative hypothesis is that absolute levels of food intake or a component of intake drive benefits observed under CR, irrespective of any energy imbalance. Previous research suggests protein may be a key macronutrient and that reduced protein intake may be responsible for benefits observed under CR [7–11].

In contrast with CR, low-protein diets are proposed to increase lifespan despite increased body fat storage related to elevated total dietary (calorie) intake under *ad libitum* (*AL*) feeding conditions in rodents [9, 10, 12–15], although very-low-protein diets and adult age at onset may alter these effects [16]. Excess body weight and body fat were associated with shorter lifespan and greater cardiometabolic disease risk in clinical and pre-clinical observations [17, 18]. Additionally, short-term adoption of CR results in acute physiologic benefits, and sustained or episodic CR following obesity establishment results in reduced body weight, decreased body fat and increased lifespan in murine models [19, 20].

Daily energy demands of endothermic organisms reflect a combination of resting, physical activity, diet-induced energy expenditure, and thermoregulation [21]. When energy intake is matched with the combined expenditure, organisms remain in energy balance. Increasing energy expenditure (e.g., via demands of thermoregulation) can result in energy imbalance when energy intake is not matched to the elevated expenditure state. Alternatively, raising the ambient temperature (T_a_) when below the lower critical temperature reduces thermoregulatory energy demands [22]. With a “pair-feeding” (PF) design, where two groups of mice are provided equal amounts and composition of food (energy and percent protein), manipulations of T_a_ can generate states of energy balance or imbalance matching that typical of CR studies (e.g., 10–40% imbalance) when performed at one T_a_.

We hypothesized that if the health benefits of CR depend on the absolute levels of energy and protein intake, then lifespan would be independent of T_a_ at a matched level of dietary intake. Conversely, if benefits from CR derive from energy imbalance, then life and healthspan benefits would be observed in organisms experiencing energy imbalance (e.g., lower T_a_ with PF), independent of the matched dietary intake. In the present set of experiments, we manipulated T_a_ to separate the effects of absolute levels of intake (calories and protein amount) from whether or not animals were feeding in energy balance. We thereby separate the impacts of energy imbalance and absolute food intake, revealing that energy imbalance is sufficient to observe the impact of CR on lifespan.

## Methods

### Overview

The contribution to this area of investigation in the current work derives from two independent sets of experiments (i.e., (1) short-term, 11-week study and (2) chronic longevity study) that were initially designed and executed by subsets of the authors under individually rigorous and controlled conditions. Through fortunate circumstances, the study leads became aware of the research groups’ independent experimental investigations and the partial replication with accompanying complementary distinction present in each study, which offered greater insight and understanding than for either set of experiments alone. Through collaborative cooperation and data sharing, the two studies were combined with accompanying statistical assessments to maximize the value and understanding of the projects. Presented below are the materials and methods for each study as performed.

### Short-term, 11-week study

#### Animals

Fifty male mice aged 6 weeks (C57BL/6 Charles River Laboratories, UK) were initially group-housed and acclimated at 21±2°C (normal housing T_a_). Throughout the study, mice were housed under 12-h light:dark cycle (on from 0600 to 1800). At 12 weeks of age, they were implanted intraperitoneally with transmitters using the VitalView telemetry and data acquisition system (MiniMitter, OR, USA; see below for body temperature and physical activity measures). Mice were single-housed following surgery and allowed to recover for 4–5 weeks. Mice were initially randomly assigned into five groups of *n*=10, and then, following principles outlined elsewhere [23, 24]*,* limited non-random reassignment was conducted to balance baseline (BL, at 17–18 weeks of age) body weight distributions across groups before placing animals in the 11-week temperature and feeding protocol. Control groups of mice were given 12-h access to *ad libitum (12AL)* food, where food was provided only in the dark phase. A 12AL feeding protocol prevents daytime eating in control animals and more closely reflects the daily feeding/fasting experiences of the restricted (PF) groups. The 12AL dark-phase feeding also removes the potential confounding effects of eating prior to culling for terminal hormonal, metabolic, and tissue measures to align controls more closely with the PF group’s feeding paradigm. The diet provided contained 10% fat, 20% protein, and 70% carbohydrate kcal (calorie: 3.85 kcal/g [16.11 kJ/g] per diet spec sheet; 4.39 kcal/g dry mass by bomb calorimetry) (D12450B, Research Diets, New Brunswick, NJ, USA). Animal work was approved by the Aberdeen Ethics Board and carried out under the Home Office Animals (Scientific Procedures) Act 1986 (License PPL/3706).

Mice were weighed between 1700 and 1800 and fed prior to the dark phase, with food removed at 0600. Over the baseline 2 weeks, the following procedures were carried out: Resting metabolic rate (RMR); glucose tolerance tests (GTT); body composition using dual-energy x-ray absorptiometry (DXA); and feces collection for bomb calorimetry and calculation of digestive efficiencies (see below).

At age 20 weeks after the final group assignment, mice were placed under treatment for a period of 11 weeks. All procedures performed during BL were repeated during the final 2 weeks of study. Following a 2-week baseline period, T_a_ was altered. Mice were housed in three separate rooms at 10°C, 21°C, and 30°C, with 21°C and 10°C utilized to induce relative energy expenditure increases for thermoregulation and compared with the thermoneutral zone housing at 30°C due to the inverse relationship between T_a_ and energy expenditure across this range. On day 1, temperatures were altered in increments of 2°C over 12 h to allow gradual adjustment.

Three of the five groups continued with 12-h *AL* feeding at each of the temperatures (AL10*—* housed at 10°C, fed 12 h *AL*; AL21—housed at 21°C, fed 12 h *AL*; AL30—housed at 30°C, fed 12 h *AL*). Of the remaining two groups, one group was housed at 21°C and pair-fed with those housed at 30°C (21(PF30) group), and the 10(PF21) group was housed at 10°C and pair-fed to those housed at 21°C. Pair-fed animals were matched to mice of similar body weight and food intake. Pair-fed intakes for the 21(PF30) and 10(PF21) groups were calculated daily. These feeding regimens continued over the remainder of the study, ~11 weeks.

Dissections were performed approximately 4 h prior to dark phase, when all mice would be in a fasted state. Mice were killed by a terminal CO_2_ overdose. Blood was taken by cardiac puncture and split into tubes containing the anticoagulants heparin or EDTA. These were kept on ice and later spun for plasma separation and frozen storage at −80°C until assays were performed.

#### Body Temperature and physical activity analysis

Minute by minute recordings from implants are transmitted via an ER-4000 receiving platform (MiniMitter, OR, USA). Daily, dark, light and food anticipatory data (3 hours pre-feeding) were calculated for analyses.

#### Body composition

Fat mass and fat-free mass were quantified using DXA (GE PIXImus2 Series Densitometers installed with software version 1.46.007; GE Medical Systems Ultrasound and BMD, UK) [25]. DXA was performed at BL and at 4 and 8 weeks during the experimental period and end of the study.

#### Glucose tolerance tests

Glucose tolerance tests (GTTs) were carried out on each animal during BL and at the end of the study (~11 weeks). GTT was carried out in a fasted state (9 h). Blood glucose was measured from tail blood using the OneTouch® Ultra® 2 glucose monitor (Johnson and Johnson, New Jersey, USA). Each mouse received an intraperitoneal injection of 2% glucose solution (Sigma, UK) equivalent to 2 mg/g body weight. Blood was sampled at exactly 15, 30, 60, and 120 min after injection, and blood glucose levels (mmol/l) were recorded.

#### Circulating biomarkers

Circulating leptin, insulin, and tumor necrosis factor (TNF-α) were measured in plasma taken at the end of the study using the Milliplex^TM^ mouse adipokine panel (MADPK-71K, Millipore, Watford, UK; intra-assay CV: <4.5%). Fasted plasma insulin-like growth factor-1 (IGF-1) was measured using a mouse-specific enzyme-linked immunosorbent assay (ELISA) (R&D Systems Europe Ltd, Abingdon, UK; intra-assay CV: <5.6%).

#### Antioxidants

Antioxidant enzyme activities in the liver were measured using direct spectrophotometric methods, as described previously [26]. Homogenates from ~50 mg of liver were prepared in ice-cold 50 mM phosphate buffer (PB, pH 7.4), centrifuged, and supernatants collected. All reactions were carried out in triplicate and absorbance read at 25°C using the SpectraMax Plus384 spectrophotometer with SoftMax software. Catalase activity was measured on the day of homogenization following the methodology of Cohen [27]. Glutathione peroxidase (GPX) activity was determined by a modification of the method of Paglia and Valentine [28]. Total superoxide dismutase (SOD) activity was measured using a method originally described by Marklund and Marklund [29], as this method cannot discriminate between the different forms of SOD (i.e., CuZn, Mn, and FE SODs). Specific antioxidant activities were calculated by adjusting for total protein per sample, and protein content was assessed using the Bradford assay [30]*.*

#### Indirect calorimetry

All measurements were made within the thermoneutral zone (30°C) in the post-absorptive state (non-feeding). Oxygen (O_2_) consumption and carbon dioxide (CO_2_) production were measured using custom-built flow-through respirometer chambers. Mice were placed in a Perspex cylinder (volume 1L), within an incubator during measurement. The respiration chamber was attached to a Xentra 4100 gas analyzer calibrated using oxygen-free nitrogen, 5% CO_2_ in nitrogen (BOC special gases), and outside surrounding air (20.95% O_2_) prior to all measurements. Each chamber was ventilated with a metered flow rate between 450 and 600 ml/min (Mass-flow controllers, MKS Instruments, Cheshire, UK) with air from the outside of the building, dried using silica gel, and measured using a calibrated Alexander Wrights Ltd precision test meter (DM3A, accurate to 0.05%). Gases were dried through silica gel prior to measurements taken every 30 s for 3 h. RMR was calculated using the Weir equation from the lowest O_2_ and CO_2_ averaged over a 5 min/10 measurement window.

#### Neuropeptides

Brain region-specific expression of neuropeptides was measured by *in situ* hybridization using ^35^S-labelled antisense riboprobes (Perkin Elmer, UK) complementary to fragments of Proopiomelanocortin gene (POMC), Neuropeptide Y receptor (NPYR), Agouti Related Neuropeptide (AgRP), and Melanocortin 4 receptor (MC4R). Autoradiographs were scanned together with an optical density scale (Stouffer Graphic Arts Equipment, Mishawaka, IN). Integrated optical density (IOD) was determined using the ImageJ software system (WinZip Computing Inc., USA). Gene expression was calculated from the mean IOD taken from anatomically matched sections from each mouse for each brain region of interest.

#### Digestive efficiency

Feces were collected over 6 days during baseline and near the end of the study. Feces were carefully separated from sawdust, weighed, and dried; a sample of diet was also weighed and dried. Gross energy content for diet or fecal sample was measured by bomb calorimetry (Parr 6100 calorimeter using a semi-micro 1109 oxygen bomb 1109A, Scientific and Medical Products Ltd, Cheadle, UK) with a minimum of three replicates, within ±0.25 kJ. Metabolizable energy intake (MEI) (kJ/day) was calculated from the gross energy intake (GEI) and energy output (energy excreted), assuming a 3% energy loss via urine [31]. The apparent energy absorption efficiency (AEAE) was calculated as the percentage of the ingested food taken up by the body.

### (Chronic) Longevity study

#### Animals

C57BL/6J male mice (*n*=160) were purchased from the Jackson Laboratory (Stock No: 000664, Bar Harbor, ME, USA) at ~6 weeks of age and were acclimated to the specific pathogen-free facility. At ~12 weeks of age, mice were randomized via a random number generator to one of the two temperature groups in a 1:1 ratio and single-housed in standard, ventilated mouse cages on an open-rack system within environmental chambers (Powers Scientific, Pipersville, PA, USA) that were set to either 22°C or 27°C. Chamber temperatures were monitored with Onset HOBO data loggers (light and temperature; UA-002-08, ONSET Computer Corp, Bourne, MA, USA) during the course of the study. Mice were maintained on a standard 12:12 light:dark cycle (0600–1800 h) with no observance of daylight saving time changes. Cages were rotated within the environmental chambers twice a week to reduce the influence of variations of sound, light intensity, etc., within the chamber shelving systems. Animal health was checked daily, and moribund animals were euthanized according to the study protocol. Study protocols were approved by the University of Alabama at Birmingham Institutional Animal Care and Use Committee (IACUC).

#### Longevity study design

**Fig. 1:**
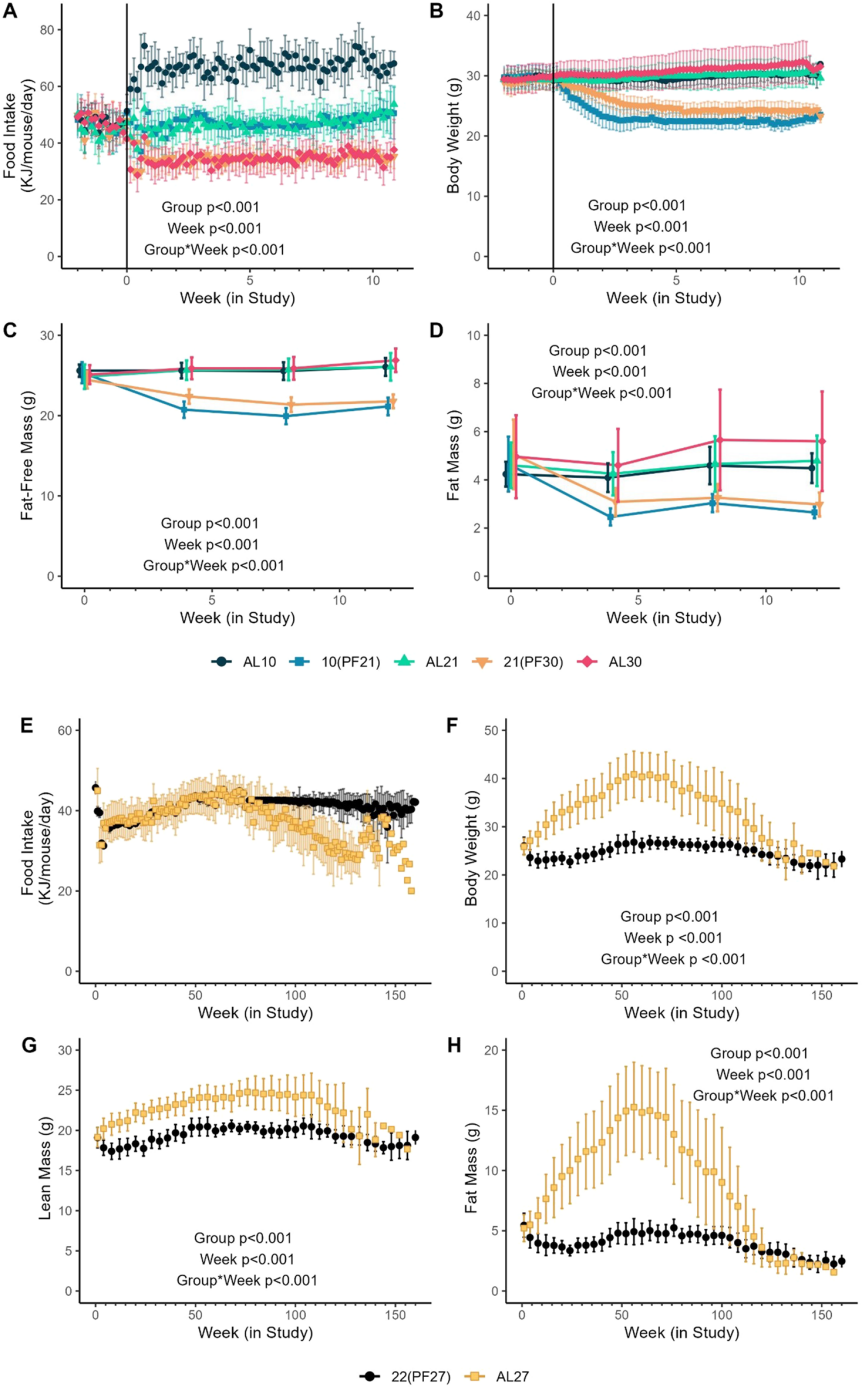
Food intake, body weight, and body composition by ambient temperature (T_a_). Short-term (**A–D**). **A** Mean (±SD) daily food intake, **B** mean (±SD) body weight, **C** mean (±SD) fat-free mass, and **D** mean (±SD) fat mass measured by DXA (AL10 [T_a_ 10°C, *ad libitum*], 10(PF21) [T_a_ 10°C, *pair-fed* to 21°C], AL21 [T_a_ 21°C, *ad libitum*], 21(PF30) [T_a_ 21°C, *pair-fed* to 30°C], and 30*AL* [T_a_ 30°C, *ad libitum*]) over ~11 weeks (20–31 weeks of age) in male C57BL/6J mice (*n*=10/group). Chronic longevity study (**E**–**H**). **E** Mean (±SD) daily food intake, **F** mean (±SD) body weight, **G** mean (±SD) lean mass, and **H** mean (±SD) fat mass measured monthly by QMR (22(PF27) [T_a_ 22°C, *pair-fed* to 27°C] or AL27 [T_a_ 27°C, *ad libitum* fed]) over the life course in male C57BL/6J mice (*n*=80/group at start, age in weeks)

Beginning at ~12 weeks of age, the C57BL/6J male mice at 27°C were provided free access (*ad libitum,* 24-h continuous) to a defined diet based on AIN-93G (65.1% kcal carbohydrate, 16.4% kcal fat, and 18.8% protein [calorie: 3.89 kcal/g; 16.28 kJ/g]; TestDiet, Richmond IN, USA), with the diet formulation switched from AIN-93G to AIN-93M (77.5% kcal carbohydrate, 9.8% kcal fat and 13.7% protein [calorie: 3.77 kcal/g; 15.77 kJ/g]; TestDiet, Richmond IN, USA) at maturity (~8 months of age). All mice in the 27°C group had food intake determined at the cage level weekly. Mean daily food intake per animal was calculated from the previous week’s totals for the 27°C animals (AL27), with the daily mean provided to the 22°C housed animals (22(PF27)) ~1 h before lights off (~1700) to encourage food intake during the normal circadian period of feeding and activity (dark phase), with any remaining food collected and weighed. Mice housed at 22°C were pair-fed through ~80 weeks of age, after which the daily provision was fixed at 2.7 g (~42.6 kilojoules, KJ) for the remainder of the study to limit the morbidity-related influence of the observed intake of the 27°C group inducing greater calorie deficit relative to healthy animal intake needs (Fig. 1A, E–H).

#### Body composition

Body weight was measured weekly throughout the course of the study. Beginning at baseline (randomization), body composition (fat and lean mass) was determined *in vivo* in all remaining live animals in all groups by quantitative magnetic resonance (EchoMRI 3-in-1, V2.1; Echo Medical Systems, Houston, TX, USA) at successive time points (~1-month intervals).

#### Lifespan

Observed lifespan (days alive) was recorded as the age when the animals died naturally or were euthanized per local IACUC protocol. Natural death or moribund status (moribund definition - body condition score of “1,” prolonged hypothermia, inability to eat/drink, self-mutilation/deformities, ataxia, inability to move, and/or respiratory distress) termination was recorded to the nearest day during daily cage checks.

#### Serum analytes

Total blood was collected from the trunk by decapitation and kept on ice for at least 15 min in 1.5 ml microtubes. Samples were centrifuged for 10 min at 3000 x *g* at 4°C, with the serum layer carefully collected. Insulin was measured using a Millipore Corporation (Billerica, MA) Sensitive Rat Insulin radioimmunoassay (RIA, #SRI-13K; intra-assay CV: 1.56%). Insulin-like growth factor-1 (IGF-1) was measured using two ALPCO (Salem, NH, USA) IGF-1 (mouse/rat) enzyme-linked immunosorbent assays (ELISA, #22-IGIMS-E01; intra-assay CV: 3.125%). Leptin was measured utilizing a Millipore Corporation (Billerica, MA, USA) Mouse Leptin ELISA kit (#EZML-82K, intra-assay CV: 1.499%). TNF-α was measured using a MesoScale Discovery (Rockville, MD, USA) Mouse TNF-α kit via chemiluminescence (one sample from 22(PF27) at 52 weeks and one from 22(PF27) at 112 weeks had insufficient volume for TNF-α measures, and data were not imputed; (#K152QWD-1, intra-assay CV: 1.8%).

#### Healthspan

Motor health scoring (ledge test, hind-limb clasping, kyphosis, gait observation) was performed cage-side using a modified motor function phenotype scoring system developed by Guyenet and colleagues during the handling of the study animals for routine measures [32]. Four measures were conducted to evaluate balance and coordination (ledge test), motor reflex (hind-limb clasping), coordination and muscle function walking on a flat surface (gait), and dorsal curvature of the spine due to loss of muscle tone of spinal muscles (kyphosis). Starting at approximately 1 year of age and continuing every ~12 weeks until the end of life, mice were assessed in each of the four measures, and each measure was repeated up to three times (replicates) during a given timepoint of measure. Assessments were conducted at the respective room temperature within hours 4 to 7 of the beginning of the light cycle.

To assess the ledge walking phenotype, mice were placed on the rim of the home cage and observed for their ability to walk along the rim of the cage without falling. A mouse capable of walking along the rim of the cage without losing balance and being able to lower itself into the cage exhibited a normal phenotype. The most severe deficit was shown by mice unable to move on the ledge, shaking or falling from the rim of the cage. The degree of hind-limb clasping was observed by lifting the mouse by the base of the tail clear of its cage and observing the mouse for 10 s. A mouse with hind limbs splayed outward exhibited a normal phenotype, but progressive retraction of hind limbs demonstrated increasing deficits in reflexes. Gait was observed for a mouse placed on a flat surface outside of its cage. For a normal gait, the mouse walked with its body weight evenly supported on all limbs and its abdomen not touching the ground. Increasing deficits were associated with signs of limps, tremors, lowered pelvis, dragging abdomen, and difficulty moving. Finally, kyphosis was assessed as either normal (no signs of curvature, easily straightened spine) to increasing deficit associated with mild dorsal curvature to persistent and pronounced curvature while sitting or walking.

### Statistical analyses

#### Longevity study

Power estimates for the longevity study were performed using the primary outcome of survival for male C57BL/6J mice from published survival data. Calculated sample sizes were estimated to provide ≥80% power to reject the null hypothesis for no difference in survival for a group pairwise comparison (*n*=80 per group). Statistical analyses were performed using SAS software (v. 9.4 for Windows, SAS Institute, Cary, NC, USA). The lifespans of the two T_a_ groups [22°C: 22(PF27) and 27°C: AL27] were compared using survival analysis by running a Cox proportional hazard regression model. The proportional hazards assumption for T_a_ group was tested and satisfied (*p*>0.05) for the Cox models [33]. Primary survival analyses were performed for all-cause mortality (as an intent-to-treat model with no censoring). The study was designed to last until all the animals died, permitting analyses by general linear models to compare the overall lifespans of the altered T_a_ groups. Percentile regression compared 50th and 90th percentile longevity between groups. Maximum lifespan based on the 90th percentile of survival was performed as described in Gao-Allison [34] and Wang-Allison [35].

#### Acute study

Energy intake differences in the *AL* groups in the short-term, 11-week study were compared using a linear mixed model between T_a_ groups with week and week*T_a_ group as additional covariates. To account for repeated measures, a random effect for animal with compound symmetry (CS) as a covariance structure was included.

#### Acute and Longevity study

For both the short-term and the chronic study, body weight between T_a_ groups was analyzed with linear mixed models with T_a_ group, week, and T_a_ group-by-week interaction as covariates. The model for the chronic study additionally included weight at baseline as a covariate (with at least 30% survival from week 1 to week 116). To account for repeated measures of these items, random effects for animal were included with a covariance structure of compound symmetry (CS) for short-term study and autoregressive AR(1) for the longevity study.

Likewise, body composition differences between groups were assessed with linear mixed models with T_a_ group, week, and T_a_ group by week interaction as covariates. The model for fat mass and lean mass in the chronic study additionally adjusted for fat mass and lean mass at baseline, respectively. In both studies, a random effect for animal was included with an AR(1) covariance structure. The four different motor health indicators (ledge test, gait, kyphosis, hind-limb clasp) in the chronic study were analyzed with linear mixed models with T_a_ group, week and T_a_ group by week interaction with a random effect for animal and AR(1) covariance structure across the week. The average of the three replications of measurements was used in the analysis.

Hormones (leptin, TNF-α, IGF-1, and insulin after log-transformation) and antioxidants (catalase, SOD, and GPX) in the 11-week (acute) study were analyzed using one-way ANOVA to detect differences by T_a_ groups. For TNF-α, 19 of the 50 animals had missing TNF-α values because they were below the detectable limit (12.2pg). Therefore, those values were imputed as 0.1 pg/ml (0.0001 ng/ml). In the chronic study, the group differences in the same set of hormones were analyzed with two-sample t-tests within the time of planned sac after log transformation.

Glucose tolerance test values were analyzed with linear mixed models at baseline and the follow-up time point (~11 weeks). We included an indicator for time (0, 15, 30, 60, and 120 min), T_a_ group, and T_a_ group by time interaction. We included a random effect for animal with a CS covariance structure. Total area under the curve (AUC) for glucose was analyzed with a linear mixed model to model change from baseline to end of the study measure.

To assess the association between age at maximum body mass (in weeks) and longevity (in weeks), a general linear model was used that included group and the interaction of group and age at maximum body mass. A similar analysis was performed for age at maximum lean mass and age at maximum fat mass on longevity. The age at maximum lean and fat mass was obtained from the monthly body composition measures, whereas the age at maximum body mass was calculated from the weekly body weight data. If an animal had the same maximum weight or lean/fat mass on two or more days, the earliest date was used in the analysis.

Statistical significance was considered at *p*<0.05, with no adjustment for multiple comparisons or multiple outcomes.

#### Data availability

Data pertaining to the findings presented in this article will be made publicly available through a repository as cited in the open data statement.

## Results

In a short-term, 11-week study, energy intake under *AL* feeding was significantly (all *p*<0.001) inversely related to T_a_ (10°C>21°C>30°C, Fig. 1A). Despite this, the body weight of AL21 (*AL* fed at T_a_ 21°C) and AL10 (*AL* fed at T_a_ 10°C) mice was not significantly different (*p*=0.855, Fig. 1B). PF mice in a cooler T_a_ based on the *AL* intake of the warmer T_a_ (specifically, 10(PF21) and 21(PF30)) resulted in rapid, significant and sustained reductions in body weight in the lower T_a_ groups compared to the *AL* groups (Fig. 1B). AL21 mice progressively increased fat-free mass, with no significant effects on fat mass (Fig. 1C and D). Warmer T_a_ (AL30) resulted in both fat and fat-free mass gains, while changes at cooler AL10 were less pronounced for either fat or fat-free mass (Fig. 1C and D). PF to warmer T_a_ resulted in rapid and significant reductions in both fat and fat-free mass in both groups (10(PF21), 21(PF30); Fig. 1C and D), with fat mass differences reflected in specific adipose depots (Fig. S1).

Consistent with the short-term study, the energy intake of AL27 mice varied over time in the longevity study, with an initial drop upon 27°C housing (lower energetic demand), followed by steady increases in energy intake to approximately 70 weeks (16 months) of age and subsequent declines coincident with morbidity related to individual animal mortality events. Food provision for the 22(PF27) group was necessarily fixed at the mean intake from ~80 weeks (Fig. 1E). Paralleling energy intake, AL27 mice gained weight during the first 52 weeks of the study, with a subsequent plateau of weight followed by a loss of weight coincident with the period of increasing mortality at later ages (Fig. 1F). The 22(PF27) mice had a transient reduction in body weight with recovery toward that of baseline and stable plateau until late in life (Fig. 1F). Overall, AL27 mice gained more weight than 22(PF27) mice fed the same food but kept under colder conditions (Fig. 1F), consistent with the short-term study.

AL27 mice achieved a higher absolute lean body mass during early life versus 22(PF27) (Fig. 1G), with significant differences in lean mass between groups observed through 116 weeks of age (Fig. 1G). While fat mass represented ~20% of total body weight at study randomization in both groups, AL27 mice gained significantly more fat mass over ~70 weeks (Fig. 1H), reaching ~37% total body fat mass and subsequently losing fat mass during the later stages of lifespan (Fig. 1H). Conversely, 22(PF27) mice lost fat mass with initial PF and thereafter presented a more blunted pattern of increase and decrease in fat mass within while never exceeding the baseline group fat mass (Fig. 1H).

**Fig. 2:**
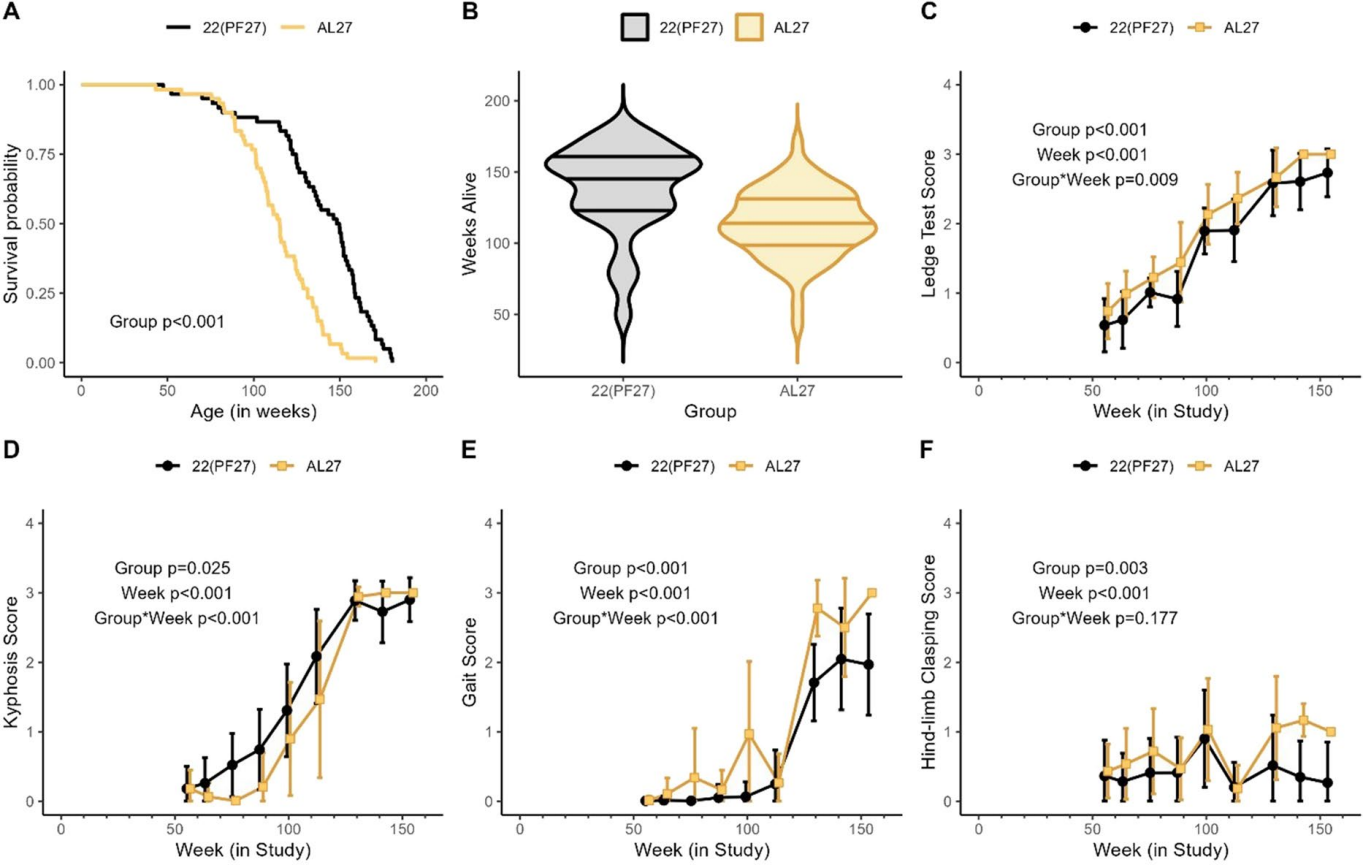
Survival and functional healthspan. **A** Kaplan-Meier survival plots and **B** violin distribution plots by group for (22(PF27) [T_a_ 22°C, *pair-fed* to 27°C] or AL27 [T_a_ 27°C, *ad libitum* fed]) in male C57BL/6J mice (*n*=60/group). Cage side battery of functional and physical assessments including **C** ledge test, **D** kyphosis, **E** gait analysis, and **F** hind-limb clasping by age at assessment (weeks 56, 64, 76, 88, 100, 113, 130, 142, 154). Scores (mean ±SD) plotted by group for (22(PF27) [T_a_ 22°C, *pair-fed* to 27°C] (starting *n*=56, decreasing with mortality) or AL27 [T_a_ 27°C, *ad libitum* fed]) (*n*=58) in male C57BL/6J mice. Higher numeric scores reflect greater impairment (**C**–**F**)

The mean lifespan of all animals combined was 126 weeks, with normal distributions of days alive between groups. Significant differences in overall survival (22(PF27)>AL27; HR=3.14; *p*<0.001; Table 1, Fig. 2A and B were observed, whereby 22(PF27) mice lived significantly longer than the AL27 mice despite the paired food and energy intake (Fig. 2A and B). The 22(PF27) mice were significantly longer lived at the 50^th^ percentile and 90^th^ percentile in percentile regression (*p*<0.001; Fig. 2A, Table 1). Maximum lifespan analysis showed that mice at 22(PF27) were significantly more likely to reach the 90th percentile (164 weeks; 1/60 in AL27 group, compared to 11/60 in 22(PF27) group; *p*=0.004 per Wang-Allison test; Table 1), as well as to live longer during old age (per Gao-Allison test, *p*=0.003).

**Table 1:** Survival analysis

		Temperature group	
		22(PF27) (*n*=60)*	AL27 (*n*=60)*
Mean survival (weeks) (Std. Dev.)		138.37 (31.23)	114.03 (23.75)
Median survival (weeks)		148.93	115.07
Range (weeks)		47.43 – 180.43	43.00 – 170.71
Cox regression			
Hazard ratio, AL27 vs. 22(PF27)	Estimate (95% CI)	3.14 (2.10, 4.68), p<.001	
Percentile regression			
50th percentile, AL27 vs. 22(PF27)	Estimate (95% CI)	−33.36 (−46.24, −20.47), *p*<.001	
90th percentile, AL27 vs. 22(PF27)	Estimate (95% CI)	−29.07 (−39.97, −18.16), *p*<.001	
Maximum lifespan analysis			
90^th^ percentile of survival (weeks)		164	
Count of animals that survived until 164 weeks		11/60	1/60
Fisher’s exact test (Wang-Allison test)^a^		*p*=.004	
Mean lifespan among survivors past 164 weeks (weeks)		172.82	170.71
Wilcoxon test (Gao-Allison test)^b^		*p*=.003	

^a^Fisher’s exact test compares the proportion of animals that survived into old age, defined as the 90th percentile across animals from both groups (164 weeks)

^b^ Wilcoxon-Mann-Whitney test compares the distributions of the variable *Z*_*i*_ defined as the survival time of animal *i* if survival time was greater than 90th percentile and 0 otherwise.

*Excluding *n*=20/group from baseline that was collected for intermediate measures at pre-defined ages

To monitor changes in functional/health biomarkers of aging in mice in the longevity study, non-invasive assessments of functional characteristics were quantified beginning at ~1 year of age and approximately quarterly thereafter. As noted in Figure 2C, D, E, and F, both groups showed increasing deficits with time/age, while AL27 mice exhibited earlier and more progressive declines in balance (ledge test), walking (gait analysis), and neurologic (hind-limb clasping) abnormalities, which were significantly higher than 22(PF27) animals. Kyphosis was observed more frequently in the 22(PF27) group versus the AL27 group, coincident with lower body weight and body fat masses (Fig. 2C, Fig. 1F and H).

Age at maximum body weight was significantly positively associated with days alive, with a significant interaction of age at maximum body weight by T_a_ group (p=0.008; Table 2). Despite the longer lifespan of the 22(PF27) group (Fig. 1F and Fig. 2A), age at maximum body weight remained significantly positively associated with lifespan among individuals within each temperature group (Table 2).

**Table 2:** Association of age at max body weight or max body fat on lifespan

	Group 22(PF27)	Group AL27	Interaction of group x age
Age (weeks) at max weight ^a^	0.23 (0.05,0.42), *p*=0.014	0.80 (0.43,1.17), *p*<0.001	*p*=.008
Age (weeks) at max lean mass ^b^	0.28 (0.09,0.47), *p*=0.005	0.76 (0.48,1.04), *p*<0.001	*p*=.006
Age (weeks) at max fat mass ^b^	0.17 (−0.02,0.37), *p*=0.086	0.88 (0.38,1.37), *p*<0.001	*p*=.010

Analysis from general linear model of age at max body weight, lean mass, fat mass on longevity, by group and group x age interaction. Coefficients represent the effects of 1-week higher age at maximum weight (maximum lean and maximum fat mass, respectively) on lifespan (in weeks) by group

a. Data for age at maximum body weight came from the weekly weight data

b. Data for age at maximum lean mass and fat mass came from monthly body composition data

Body composition analysis revealed a significant positive relationship between age at maximum lean mass and longevity across all animals, as well as within each group (Table 2). This age at maximum lean mass with lifespan relationship was significantly larger in the AL27 group compared to the 22(PF27) group. Similarly, and despite a smaller gain in total fat mass in the 22(PF27) group, a significant positive relationship between age at maximum fat mass and longevity was also observed within the AL27 group, with a weaker relationship in the 22(PF27) group, which had less variability in fat mass (Fig. 1, Table 2).

**Fig. 3:**
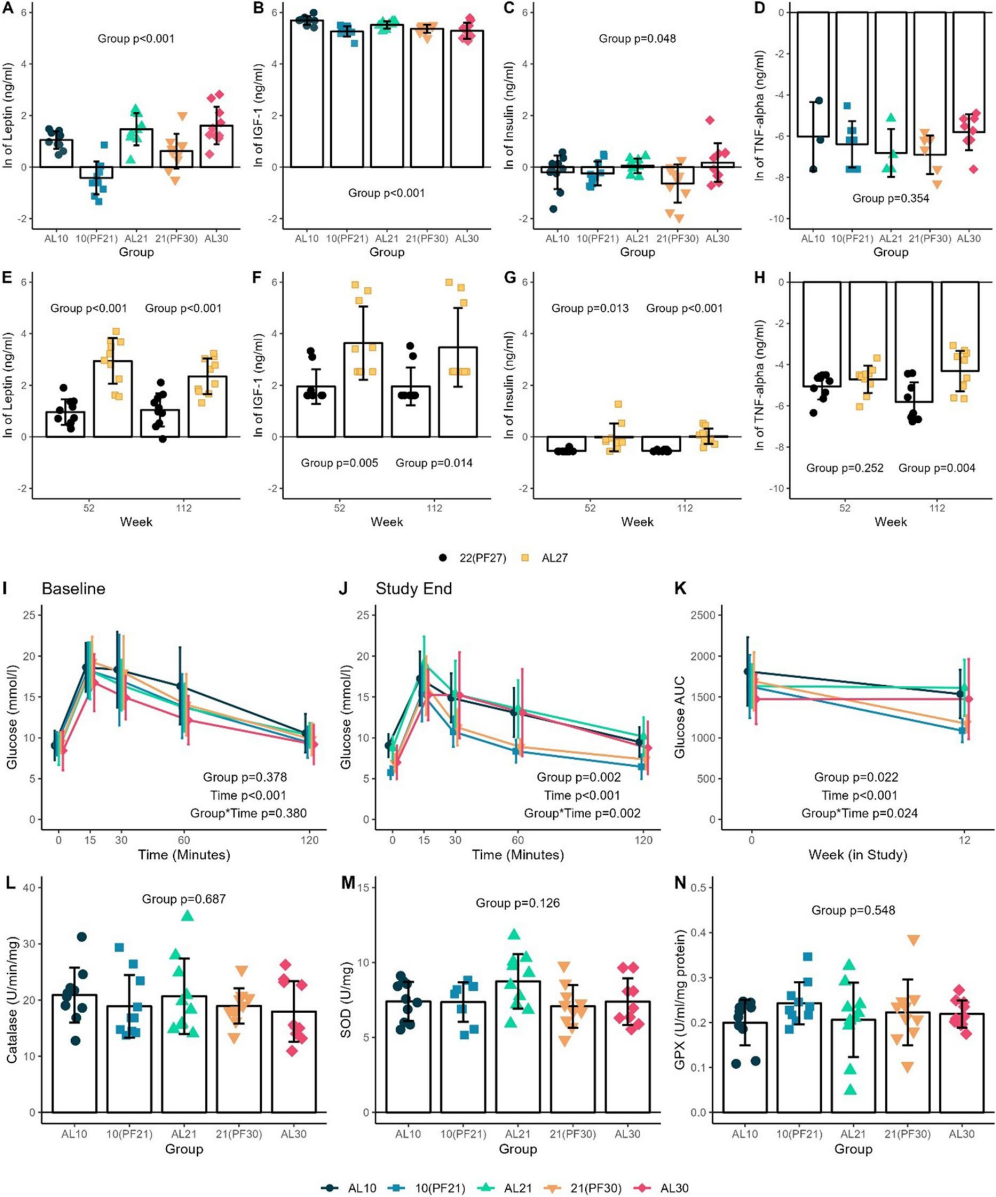
Biomarkers, metabolites, and antioxidants. **A** Leptin (ln transformed), **B** insulin-like growth factor 1 (IGF-1, ln transformed), **C** insulin (ln transformed), and **D** tumor necrosis factor (TNF-α, ln transformed)) circulating levels at termination (11-week feeding) in male C57BL/6J mice (short-term study; *n*=10/group, with TNF-α imputation for values below detectable limit; only measured values shown; mean±SD). **E** Leptin (ln transformed), **F** IGF-1 (ln transformed), **G** insulin (ln transformed), and **H** TNF-α (ln transformed) circulating levels by group **22(PF27)** [T_a_ 22°C, *pair-fed* to 27°C] or AL27 ([T_a_ 27°C, *ad libitum* fed]) at ~52 weeks (1 year) and ~112 weeks (~50% tile of survival) of age in male C57BL/6J mice (*n*=10/group/timepoint, except 9 for 22(PF27) 52/112 weeks; mean ±SD by group; chronic longevity study). Glucose tolerance. **I** Glucose tolerance test at baseline (before T_a_ randomization), **J** after ~11 weeks feeding, and **K** AUC between baseline and ~11-week follow-up among groups at study completion in male C57BL/6J mice (*n*=10/group; mean ±SD; Short-term study)**.** Antioxidants. **L** Catalase (U/min/mg), **M** glutathione peroxidase (GPX, U/ml/mg protein), and **N** superoxide dismutase (SOD, U/mg) levels among groups (AL10 [T_a_ 10°C, *ad libitum* fed], 10(PF21) [T_a_ 10°C, *pair-fed* to 21°C], AL21 [T_a_ 21°C, *ad libitum* fed], 21(PF30) [T_a_ 21°C, *pair-fed* to 30°C] and AL30 [T_a_ 30°C, *ad libitum* fed]) at termination in male C57BL/6J mice (*n*=10/group; mean ±SD; short-term study)

Evaluating effects of circulating biomarkers in short-term study samples revealed significant differences among T_a_ groups for leptin (*p*<0.001), insulin-like growth factor 1 (IGF-1, *p*<0.001) and insulin (*p*=0.048), but not tumor necrosis factor (TNF-α) (*p*=0.354; Fig. 3 A–D); fat mass adjusted for leptin and censoring an obese outlier for insulin negated statistical significance (leptin: *p* =0.169, insulin: *p*=0.077), while fat-free mass adjusted for IGF-1 did not change the observed effect (*p*<0.001; adjusted analyses not shown). Pair-feeding at lower T_a_ ((10(PF21) and 21(PF30)) resulted in significantly lower levels of leptin compared to warmer *AL* T_a_ groups, while IGF-1 was significantly higher in the 10AL compared with the 21(PF30), and insulin levels were similar, with no significant differences in TNF-α (Fig. 3). To assess changes in biomarkers observed during chronic energy imbalance conditions and advancing age, a random subset of animals from the longevity cohorts of the T_a_ groups (*n*=10/group at ~12 months/52 weeks and 50% survival, ~27 months/112 weeks) were measured for similar outcomes. There were significantly lower levels of leptin, insulin, and IGF-1 at both time points, while TNF-α levels were reduced at 27 months in 22(PF27) relative to AL27 mice (Fig. 3 E–H). Leptin levels remained significantly different after adjusting for fat mass only at 27 months while adjusting for lean mass resulted in no significant differences in IGF-1 levels at either time point (Fig. 3. E–H; adjusted analyses not shown).

Assessments of metabolic responsiveness via glucose tolerance test in the short-term study showed no significant differences in glucose area under the curve (AUC) at the end of the study among T_a_ groups fed *AL,* but glucose levels were significantly lower at 30, 60, and 120 min and AUC at the 11-week follow-up time point for 10(PF21) compared with AL21 (Fig. 3I–K). There were no significant differences between these same groups in antioxidant capacity based on liver catalase, superoxide dismutase, or glutathione peroxidase levels (Fig. 3L–N).

**Fig. 4:**
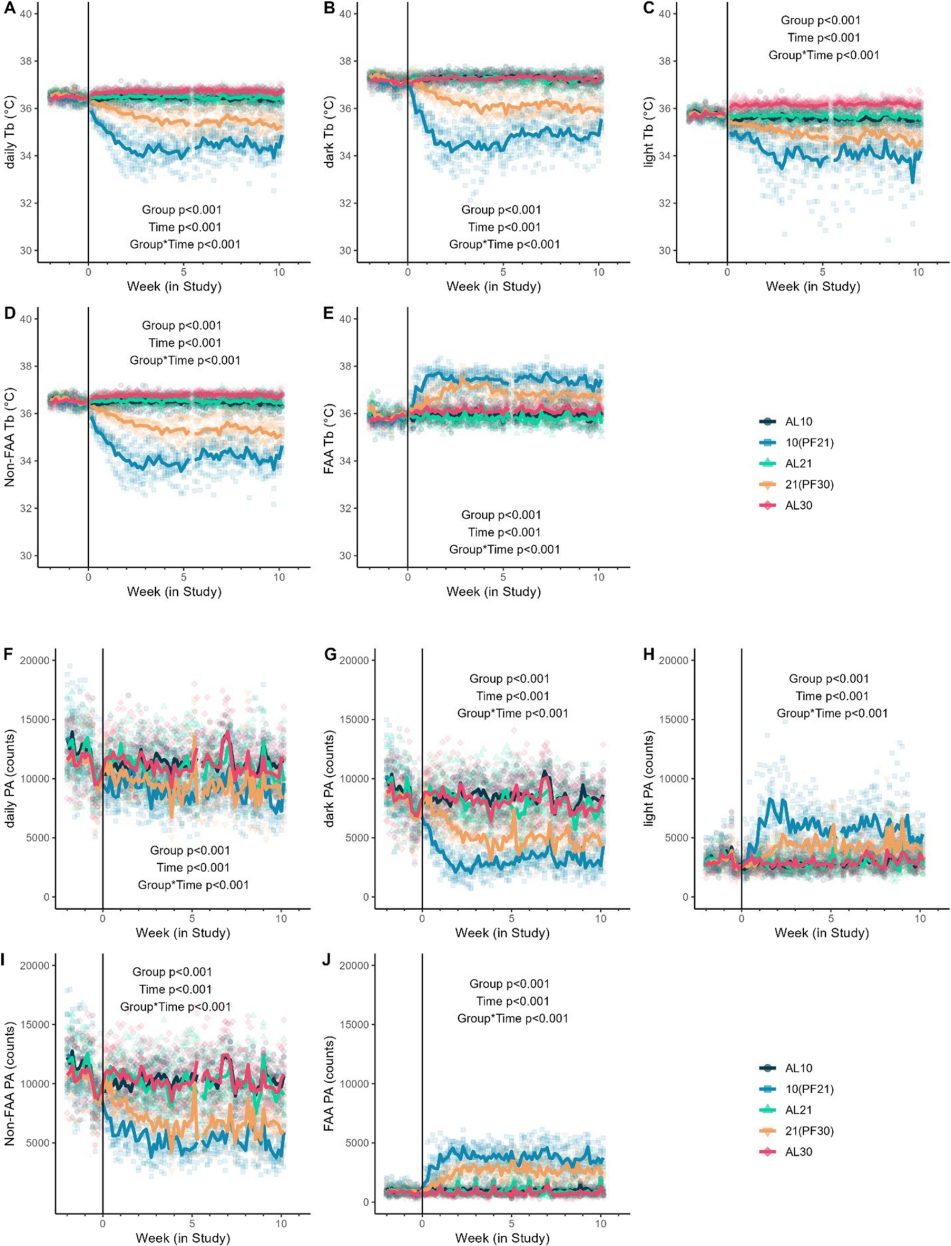
Core body temperature (T_b_) and physical activity (PA). **A** Daily mean T_b_, **B** dark-phase mean T_b_, **C** light-phase mean T_b_, **D** non-food anticipatory activity (non-FAA) mean T_b_, and **E** FAA mean T_b_. **F** Daily mean PA counts, **G** dark-phase mean PA counts, **H** light-phase mean PA counts, **I** food anticipatory activity (FAA) mean counts, and **J** non-FAA mean counts in male C57BL/6J mice (*n*=10/group; short-term study). (AL10 [T_a_ 10°C, *ad libitum* fed], 10(PF21) [T_a_ 10°C, *pair-fed* to 21°C], AL21 [T_a_ 21°C, *ad libitum* fed], 21(PF30) [T_a_ 21°C, *pair-fed* to 30°C], and AL30 [T_a_ 30°C, *ad libitum* fed]) in male C57BL/6J mice (*n*=10/group; short-term Study)

With the pair-feeding design and T_a_ differential housing, body temperature (T_b_) measures collected each minute via implanted telemetry revealed significant differences among groups. Daily averages were significantly different between all pairs of groups averaging across all time points (with the lowest in the two PF groups: 10(PF21)<21(PF30), Fig. 4A-E) with the exception of AL10 and AL21, which did not differ from each other (Fig. 4A). T_b_ in the light phase was significantly different among all groups (Fig. 4C), as was T_b_ during food anticipatory activity (FAA, Fig. 4E). T_b_ during the dark phase reflected the differences in T_a_ with energy intake (*AL* groups not significantly different from each other, but significantly higher versus PF; Fig. 4B) with non-FAA T_b_ measures significantly different for all groups except between AL10 and AL21 (Fig. 4D). These T_b_ differences were observed despite no significant among-group differences in measures of O_2_ consumption (*p*=0.885), CO_2_ production (*p*=0.821), or resting metabolic rate (*p*=0.869), even after adjusting for body weight mass differences at baseline among treatment groups (Fig. S2). Physical activity measures further highlighted significant differences among groups, with lower T_a_–housed PF groups significantly less active in the dark phase than their *AL* counterparts but significantly more active in the light phase, particularly during the FAA period just prior to lights out (Fig. 4F–J). These patterns of FAA were associated with Agouti-related protein (AgRP) and Proopiomelanocortin protein (POMC), which were significantly different among groups (overall group *p*<0.001 and *p=*0.022, respectively) in the arcuate nucleus of the hypothalamus, while melanocortin 4 receptor (MC4R, periventricular nucleus) and neuropeptide Y receptor (NPYR, arcuate nucleus nor dentate gyrus) were not significantly different among groups (Fig. S3).

With periods of feeding and fasting, which can differ between *AL* and PF groups, measures of digestive efficiency were compared. Energy intake was inversely associated with T_a_ and *AL* intake, with no difference between PF groups as designed (Fig. S4A). There was a similar pattern of energy excretion (Fig. S4C). This accompanied a significant difference in gross energy (GE) of feces among groups, with increasing GE content at increasing T_a_ (Fig. S4B). Incorporating these energetic variables revealed no significant differences among groups in overall Apparent Energy Assimilation Efficiency (AEAE, Fig. S4).

## Discussion

Multiple studies over the last decade have converged on questions of total energy versus macronutrient consumption as modifiable targets for improving health and longevity. In the current experiments, consuming matched calories with the same diet composition (macronutrient percentages) at different T_a_ revealed that energy imbalance resulting from lower T_a_ housing resulted in better maintenance of physical functioning, metabolic biomarkers, and longevity. Thus, energy balance (energy intake minus energy expenditure) was the primary contributor to the benefits observed with dietary exposure. Because total caloric and protein intake were matched in the longevity study, our results establish that an energy imbalance is sufficient to confer dietary restriction benefits on longevity: low-protein consumption was not necessary to confer the benefits (12). Unlike the low-protein diets that have been previously reported to increase longevity while inducing hyperphagia and obesity phenotypes [9, 10, 15], the present results with matched intake and improved body composition outcomes (at the group level with lower body fat) were associated with the increased longevity. These body composition improvements with lower T_a_ exposure in PF were accompanied by lower IGF-1 and insulin levels, which have been consistently implicated in longevity regulation through dietary and genetic intervention studies but were previously shown to be differentially regulated by calorie versus protein restriction [3, 26, 36]. The lower body fat and improved circulating hormone profiles align more closely with the “clean cupboards” hypothesis of CR whereby limited dietary energy resources could induce a remodeling of cellular metabolism with favorable outcomes for tissues and, ultimately, the function and longevity of the organism [5]. As observed herein, the lower T_a_ group not only lived longer but also maintained functional capacity with advancing age.

Related to whole organism metabolism, the short-term study revealed a significant difference in T_b_ with lower T_a_ and PF. This was despite no significant differences in resting metabolic rate among groups when tested at thermoneutrality. Beyond observational associations of lower T_b_ with increased longevity, genetic manipulation to reduce T_b_ resulted in extended longevity, while lower T_b_ has been implicated in the longevity and cancer-protective responses of energy-restricted feeding [1, 37–40]. Furthermore, T_b_ was a mediating factor for lifespan determination, rather than metabolic rate, under thermal conditions at the upper end of thermoneutrality [41]. The absence of a significant metabolic rate difference or antioxidant levels challenge the proposed contribution of either mechanism to the CR response [1, 42]. However, further measures later in life would be needed to confirm this.

While the energy imbalance induced in the current study via lowering T_a_ bypasses potential impacts of secondary unintended effects with exercise or pharmaceutical agents, the data raise additional questions about the extent to which additional agents could or are being utilized to tip the energy imbalance in favor of an improved metabolic and physiologic profile as was observed. This may be particularly true for interventions that reduce body fat, improve glucose tolerance and insulin signaling, and promote long-term functional maintenance. However, data from pharmaceutical agents such as sibutramine suggest more specific regulatory factors are needed to recapitulate the benefits of energetic imbalance [43]. It would be interesting to see whether more powerful appetite suppressants, such as the current generation of GLP-1 analogs, might result in sufficient energy imbalance to be beneficial in the long term for these same outcomes.

We observed a relationship between energy imbalance with lower T_a_, and the PF design resulted in low body weight, fat, and temperature and altered hormonal profiles; yet, at the individual animal level, age at max weight, lean mass, or fat mass were positive predictors of longevity. Fat mass maintenance under CR was previously shown to be a positive predictor of longevity benefits, albeit those studies focused on strain-specific responses to CR within a cohort of recombinant inbred lines versus at the individual animal level, as we report [44].

The selection of T_a_s used in the present studies ranged from the thermoneutral zone for mice to a much colder T_a_, significantly below the normal laboratory T_a_ (20–23°C), sufficient to result in a significant increase in energy expenditure to maintain thermal homeostasis (coincident with increased energy intake under *AL* feeding). The opportunity to combine data from two independently designed and performed studies that complemented each other overall with multiple similarities, yet distinct differences in individual specifics have been described. Importantly, the PF design, utilization of two different study sites for acute and chronic study comparison, use of standardized diets and daily feed provision, and use of single-housed mice and controlled environmental conditions provide good support for the observations noted. Limitations such as testing only one diet and sex, the number of longevity groups that could be performed, potential interactions of age at onset for T_a_ exposure, circadian influences related to daily feeding and potential stress effects of single housing conditions, a necessity for the study measures, are factors to consider in future work.

Despite the above considerations, these results provide strong evidence that dietary energy intake alone is unnecessary for predicting the health and longevity benefits of sustained dietary interventions. These data offer support for the implications and significance of integration of daily energy balance with modifying dietary exposure, highlighting the role that energy expenditure can have on varied phenotypes of physiological significance in aging processes.

### Supplementary information

ESM 1 (DOCX 760 kb)

## Data Availability

Data pertaining to the findings presented in this article will be made publicly available through a repository as cited in the open data statement.
