# Supplementary material for: Benefits of calorie restriction in mice are mediated via energy imbalance, not absolute energy or protein intake
Source: GeroScience. 2024 Jun 8;46(5):4809–26. doi: 10.1007/s11357-024-01166-4 (PMC11336014; doi:10.1007/s11357-024-01166-4)
Supplement: Supplementary file 1 — (DOCX 760 kb) [file 11357_2024_1166_MOESM1_ESM.docx]

Supplementary Information

Benefits of calorie restriction in mice are mediated via energy imbalance, not absolute energy or protein intake

Daniel L. Smith Jr^1-5†^, Sharon E. Mitchell^6†^, Maria S. Johnson^1-2,4-5^, Victoria K. Gibbs^1,7^, Stephanie Dickinson^9^, Beate Henschel^9^, Rui Li^9^, Kathryn A. Kaiser^2,8^, Daniella E. Chusyd^1,10^, Andrew W. Brown^11,12^, David B. Allison^1-4,9*^, John R. Speakman^6,13-15*^, Tim R. Nagy^1-5*^

**Affiliations:**

^1^Department of Nutrition Sciences, University of Alabama at Birmingham, Birmingham AL, USA;

^2^Nutrition Obesity Research Center, University of Alabama at Birmingham, Birmingham AL, USA;

^3^Integrative Center for Healthy Aging, University of Alabama at Birmingham, Birmingham AL, USA;

^4^Nathan Shock Center of Excellence in the Biology of Aging, University of Alabama at Birmingham, Birmingham AL, USA;

^5^Diabetes Research Center, University of Alabama at Birmingham, Birmingham AL, USA;

^6^School of Biological Sciences, University of Aberdeen, Aberdeen Scotland, Scotland UK;

^7^Department of Clinical and Diagnostic Sciences, University of Alabama at Birmingham AL, USA;

^8^Department of Health Behavior, University of Alabama at Birmingham, Birmingham AL, USA;

^9^Department of Epidemiology and Biostatistics, School of Public Health-Bloomington, Indiana University, Bloomington IN, USA;

^10^Department of Environmental and Occupational Health, School of Public Health-Bloomington, Indiana University, Bloomington IN, USA;

^11^Department of Applied Health Science, Indiana University School of Public Health-Bloomington, Bloomington, IN, USA

^12^Department of Biostatistics, University of Arkansas for Medical Sciences, Little Rock, AR, USA; Arkansas Children’s Research Institute, Little Rock, AR, USA

^13^Shenzhen Key Laboratory for Metabolic Health, Center for Energy Metabolism and Reproduction, Shenzhen Institutes of Advanced technology, Chinese Academy of Sciences, Shenzhen, China;

^14^Institute of Health Sciences, China Medical University, Shenyang, Liaoning, China

^15^Institute of Genetics and Developmental Biology, Chinese Academy of Sciences, Beijing, China

^†^These authors contributed equally to this work

*Corresponding Author: Tim R. Nagy: [timrnagy@ymail.com](mailto:timrnagy@ymail.com)

John R. Speakman: [j.speakman@abdn.ac.uk](mailto:j.speakman@abdn.ac.uk) ; David B. Allison: [allison@iu.edu](mailto:allison@iu.edu)


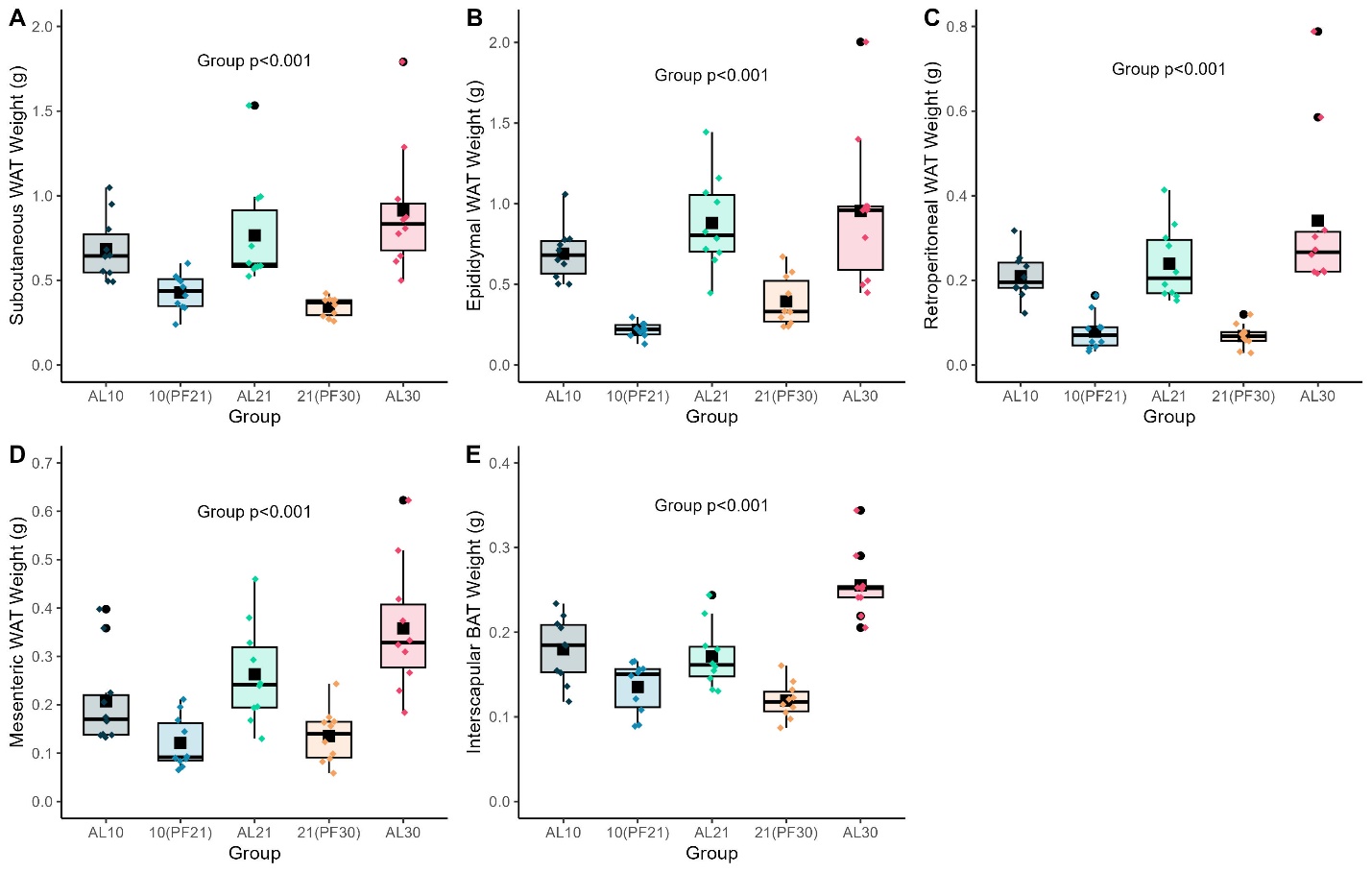
Fig. S1.

**Dissected white and brown adipose tissue depots by group ambient temperature (T_a_). Short-term Study (A–E).** A) Mean subcutaneous (Sub_Cut) white adipose tissue, B) mean epidydimal (EPI) white adipose tissue, C) mean retroperitoneal (Retro) white adipose tissue, D) mean mesenteric white adipose tissue, and (E) mean interscapular brown adipose tissue (BAT) wet weight measured at dissection in grams (**AL10** [T_a_ 10ºC, *ad libitum*], **10(PF21)** [T_a_ 10ºC, *pair-fed* to 21ºC], **AL21** [T_a_ 21ºC, *ad libitum*], **21(PF30)** [T_a_ 21ºC, *pair-fed* to 30ºC] and **30*AL*** [T_a_ 30ºC, *ad libitum*]) ~31 weeks of age) in male C57BL/6J mice (n=10/group). Boxplots showing 1^st^-3^rd^ quartile, with median line; black squares show means, black circles identify outliers.


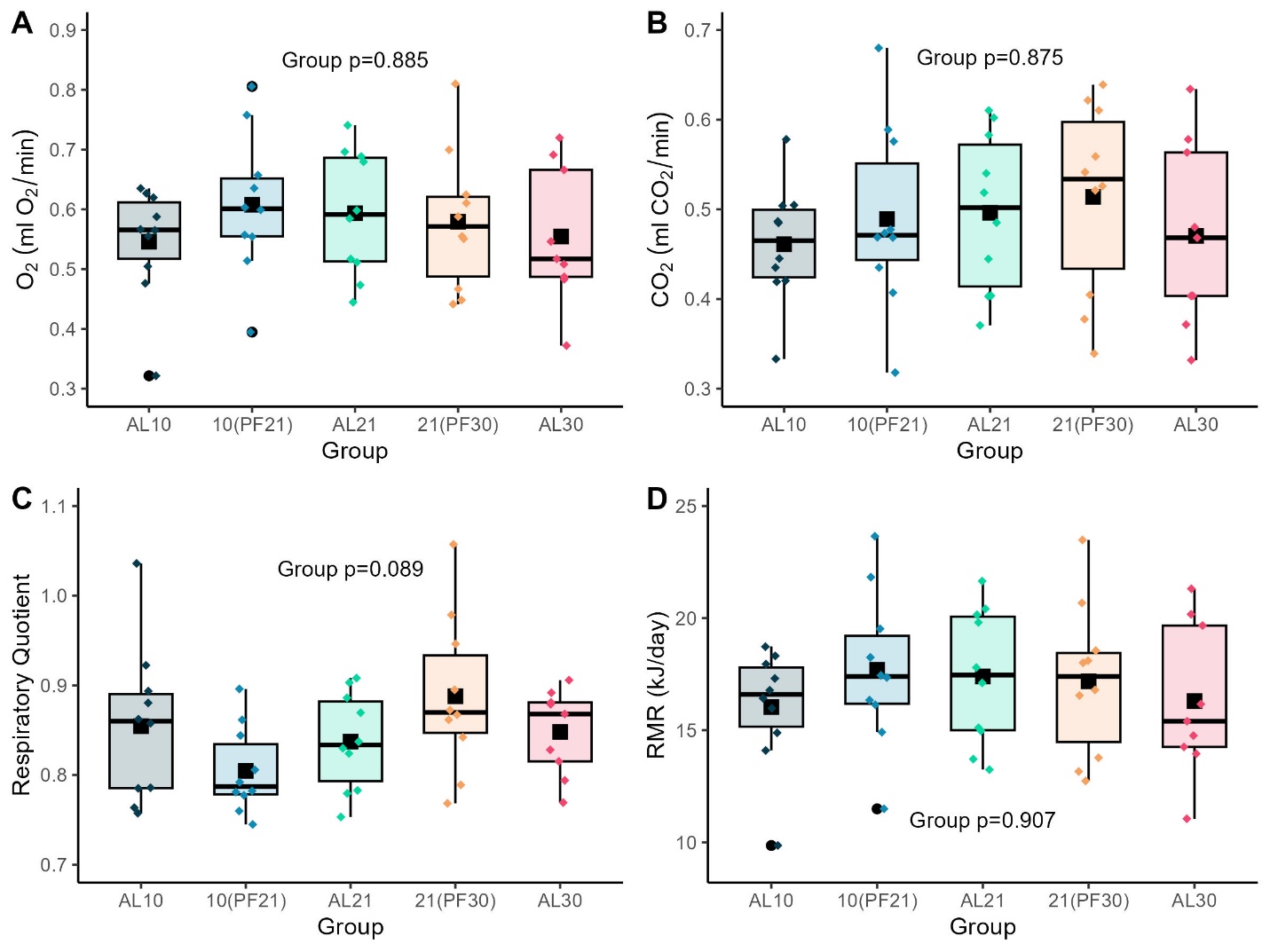


Fig. S2.

**Metabolic rate measures. Short-term Study (A–D).** A) Mean oxygen consumption (O_2_, ml/min), B) mean carbon dioxide (CO_2_, ml/min) production, C) mean respiratory quotient (RQ), and D) mean resting metabolic rate (RMR, in kJ) by group at final measure (**AL10** [T_a_ 10ºC, *ad libitum*], **10(PF21)** [T_a_ 10ºC, *pair-fed* to 21ºC], **AL21** [T_a_ 21ºC, *ad libitum*], **21(PF30)** [T_a_ 21ºC, *pair-fed* to 30ºC] and **30*AL*** [T_a_ 30ºC, *ad libitum*]) ~31 weeks of age) in male C57BL/6J mice (n=10/group). Boxplots showing 1^st^-3^rd^ quartile, with median line; black squares show means, black circles identify outliers.


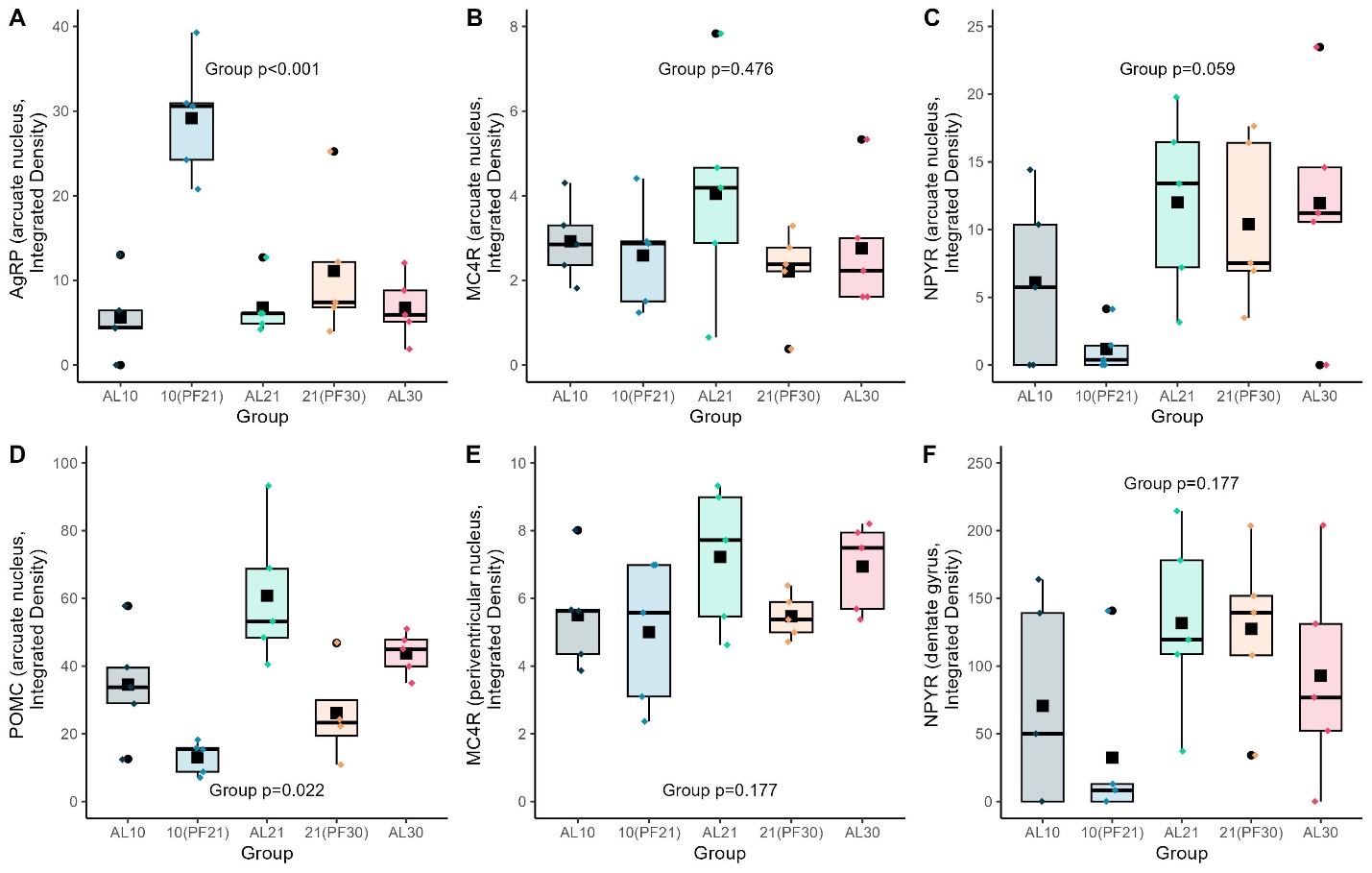


Fig. S3.

**Brain region specific expression of neuropeptides measured by *in situ* hybridization**. **Short-term Study (A–F). A)** Agouti Related Neuropeptide (AgRP) in the arcuate nucleus of the hypothalamus (Arc), **B)** Melanocortin 4 receptor (MC4R) in the arcuate nucleus of the hypothalamus **C)** Neuropeptide Y receptor (NPYR) in the arcuate nucleus of the hypothalamus, **D)** Proopiomelanocortin gene (POMC) in the arcuate nucleus of the hypothalamus, **E)** Melanocortin 4 receptor (MC4R) in the periventricular nucleus (PV) and **F)** Neuropeptide Y receptor (NPYR) in the dentate gyrus (DG) among groups (**AL10** [T_a_ 10ºC, *ad libitum* fed], **10(PF21)** [T_a_ 10ºC, *pair-fed* to 21ºC], **AL21** [T_a_ 21ºC, *ad libitum* fed], **21(PF30)** [T_a_ 21ºC, *pair-fed* to 30ºC] and **AL30** [T_a_ 30ºC, *ad libitum* fed]) at termination in male C57BL/6J mice (n=10/group; Short-term Study). Boxplots showing 1^st^-3^rd^ quartile, with median line; black squares show means, black circles identify outliers.


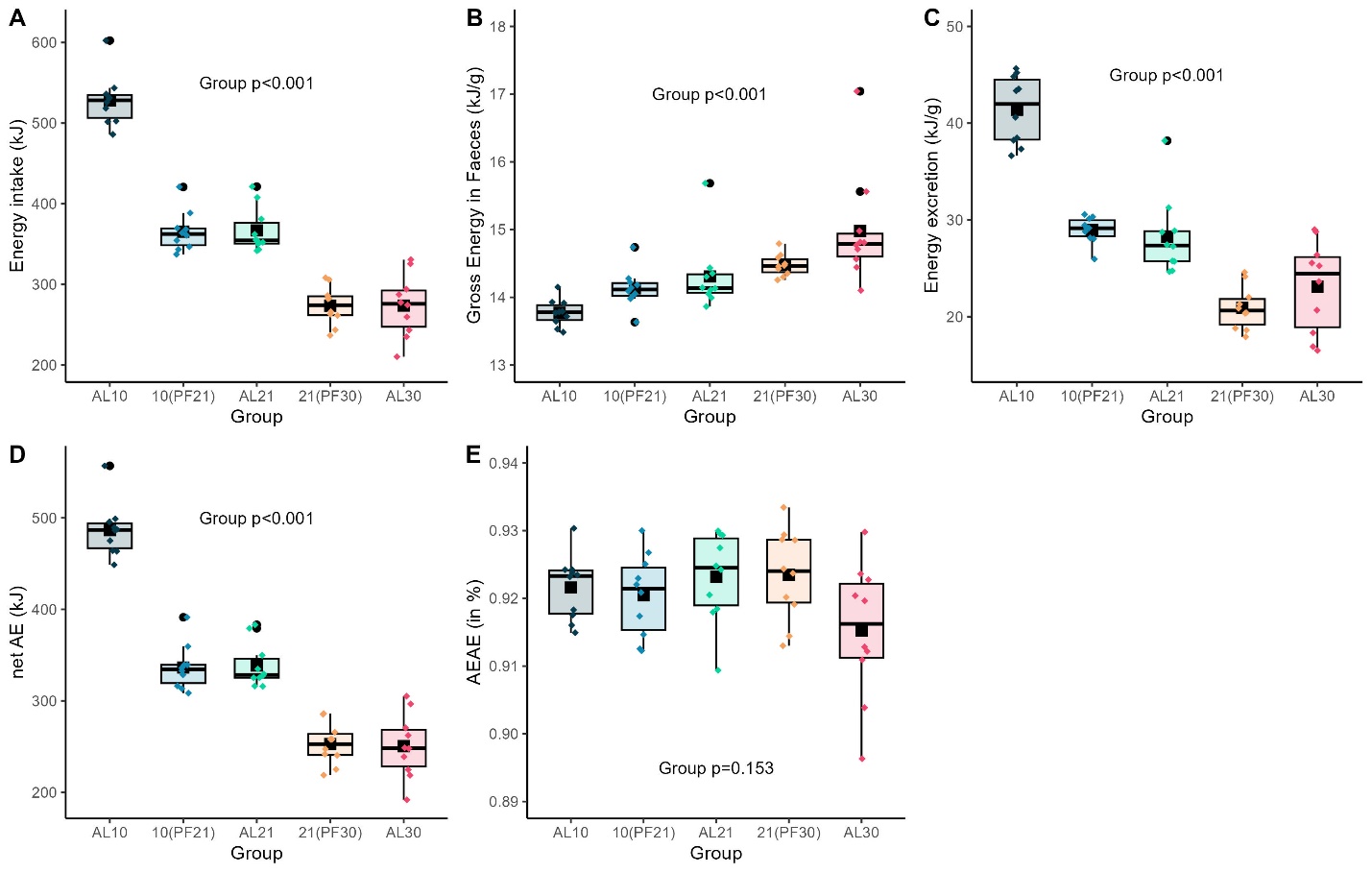


Fig. S4.

**Digestive efficiency.** **Short-term Study (A–E).** A) Mean energy intake (kJ/day), B) mean gross energy in feces (kJ), C) mean energy excretion (kJ), D) mean net assimilated energy (AE, kJ) and E) mean apparent energy assimilation efficiency % (AEAE, kJ/gram) by group at final measure (**AL10** [T_a_ 10ºC, *ad libitum*], **10(PF21)** [T_a_ 10ºC, *pair-fed* to 21ºC], **AL21** [T_a_ 21ºC, *ad libitum*], **21(PF30)** [T_a_ 21ºC, *pair-fed* to 30ºC] and **30*AL*** [T_a_ 30ºC, *ad libitum*]) ~31 weeks of age) in male C57BL/6J mice (n=10/group). Boxplots showing 1^st^-3^rd^ quartile, with median line; black squares show means, black circles identify outliers.
